# Differential regulation of monocyte oxidative burst by isoniazid in healthy and latent tuberculosis-infected subjects

**DOI:** 10.3389/fphar.2026.1763287

**Published:** 2026-03-31

**Authors:** Josephine Gal, Martina Sönnerbrandt, Clara Braian, Thomas Schön, Robert Blomgran

**Affiliations:** 1 Division of Inflammation and Infection, Department of Biomedical and Clinical Sciences, Linköping University, Linköping, Sweden; 2 Department of Infectious Diseases, Linköping University Hospital and Kalmar County Hospital, Linköping University, Linköping, Sweden

**Keywords:** classical monocytes, eosinophils, isoniazid (INH), latent tuberculosis infection (LTBI), neutrophils, pharmaceutical off-target effect, reactive oxygen species (ROS)-production, whole blood ROS-assay

## Abstract

**Introduction:**

Isoniazid (INH), a first-line drug for tuberculosis, exerts bactericidal effects through inhibition of mycolic acid synthesis. However, its potential to modulate host immunity remains unclear. Reactive oxygen species (ROS) are critical antimicrobial effectors produced by innate immune cells, and their regulation is essential for effective immune signalling and pathogen clearance, whereas excessive ROS can contribute to inflammation and tissue damage. This duality makes it important to determine whether INH modifies ROS production in innate immune cells, particularly in individuals with latent infection.

**Methods:**

We investigated whether INH affects ROS production in circulating immune cells and plasma cytokines in healthy controls and LTBI individuals before treatment initiation (n = 9 per group). Whole blood was incubated with INH at plasma concentrations observed in humans (2, 4.5, and 10.5 μg/mL). Intracellular ROS production in neutrophils, eosinophils, and monocytes was quantified using flow cytometry with the oxidation-sensitive probe DHR-123, following stimulation with fMLP, *Escherichia coli*, or PMA. IL-1β, IL-6, IL-8, TNF, IFN-γ, IL-10, and TGF-β1 were measured in INH-exposed unstimulated blood by cytometric bead array.

**Results:**

In healthy controls, INH induced a significant reduction in ROS production in monocytes (median values of DHR+ classical monocytes after *E. coli* stimulation with 0 μg/mL INH were 26.2%, 2 μg/mL INH 19.9% (p < 0.05), 4.5 μg/mL INH 16.2% (p < 0.01), and 10.5 μg/mL INH 16.3% (p < 0.01)). In contrast, INH had no effect on ROS production in LTBI individuals, who overall displayed significantly lower ROS responses to stimulation compared with healthy controls, particularly in *E. coli*-stimulated eosinophils (p < 0.0001 all data ± INH aggregated) and PMA-stimulated classical monocytes (p < 0.0001 all data ± INH aggregated). INH did not alter cytokine levels in unstimulated blood after 24 h.

**Discussion:**

These findings suggest that INH dose-dependently downregulates monocyte ROS production in healthy individuals, whereas LTBI individuals exhibit an diminished ROS response compared to healthy controls that is not further affected by INH. This work provides new insight into the immunomodulatory properties of INH and highlights the need to consider host responses, such as ROS production, in optimizing TB treatment and adjunctive therapy development.

## 1 Introduction

Tuberculosis (TB), caused by *Mycobacterium tuberculosis* (Mtb), remains one of the world’s most persistent infectious diseases. Approximately one-quarter of the global population is estimated to harbour latent tuberculosis infection (LTBI) (WHO, 2023). Following infection, the risk of developing active TB is highest within the first 2 years, around 5%, before declining substantially over time (Menzies et al., 2018). Progression to active disease is mainly dependent on host immunity, the strongest support coming from the 5 to 10-fold higher progression risk in HIV-coinfected individuals compared with immunocompetent hosts. Before the advent of effective antibiotics in the mid-20th century, TB was a leading cause of death, responsible for up to one in four fatalities in parts of Europe and North America (Barberis et al., 2017). Today, preventive regimens can prevent 85%–90% of latent infections from progressing to disease when given, but most LTBI cases in endemic areas remain untreated (Palanivel et al., 2023; Yoopetch et al., 2023; Kim et al., 2023). While these antibiotics were originally selected for their potent bactericidal activity, accumulating evidence suggests that they can also influence host immune cell function. Among them, isoniazid (INH) remains a cornerstone of both drug susceptible active and latent TB treatment.

INH enters *M. tuberculosis* by passive diffusion and is activated by the catalase-peroxidase enzyme KatG, generating reactive metabolites including reactive oxygen species (ROS). One of these metabolites, the isonicotinoyl radical, covalently binds NAD and inhibits the enoyl-ACP reductase InhA, a key enzyme in mycolic acid biosynthesis. This inhibition disrupts cell wall formation and leads to bacterial death. In addition, the ROS generated during INH activation may damage DNA, lipids, and proteins, contributing to its bactericidal activity (dos Santos et al., 2017). Although the primary antimicrobial targets of INH are relatively well defined, its potential immunomodulatory effects on host immune cells remain far less understood. Evidence suggests that INH can alter immune cell function in a variety of contexts. For example, exposure of isolated rat liver mitochondria to INH caused impairment of the electron transport chain, leading to increased ROS production (Ca et al., 2020). In Mtb-infected macrophages, INH treatment significantly activated cellular and mitochondrial ROS and enhanced autophagy, while also dampening Mtb-induced proinflammatory responses (Kim et al., 2012). In human granuloma models, INH reduced bacterial survival without affecting TNF or IL-1β production, indicating that its antimicrobial activity does not necessarily modify host-mediated cytokine responses (Yamashiro et al., 2016). Neutrophils exposed to INH showed attenuated oxidative burst at supratherapeutic concentrations, although phagocytic activity remained unaffected (Kielland et al., 2011). Taken together, these studies indicate that INH can modulate diverse immune pathways, including ROS production, autophagy and cytokine responses in a context- and cell type-dependent manner. However, these findings involve very different biological systems; ranging from innate immune cells to non-immune models such as isolated mitochondria and therefore represent heterogeneous immunological contexts. Moreover, many rely on non-physiological INH concentrations, leaving its immunomodulatory effects in human immune cells largely unresolved.

Neutrophils, eosinophils, and monocytes are critical for the early innate immune response against Mtb, shaping the outcome of infection together with the specificity of the adaptive immune response. Classical monocytes (CD14^+^CD16^−^), the dominant monocyte subset, are key early responders in TB, in part through their strong capacity for ROS production (Sampath et al., 2018; Balboa et al., 2015). ROS are early effectors produced by phagocytes, generated through mitochondrial and NADPH oxidase pathways as part of the oxidative burst, which contributes to restricting bacterial growth (Araújo-Pereira and Andrade, 2025). Eosinophils, though less studied in TB, also produce ROS and release cytotoxic mediators, suggesting a potential role in modulating both host defence and immunopathology. ROS also act as signalling mediators that stimulate antimicrobial processes such as autophagy and apoptosis. However, excessive ROS can initiate inflammatory signalling and multiple necrotic cell death pathways, thereby exacerbating tissue damage and promoting disease progression (Amaral et al., 2019; Amaral et al., 2022). The balance between these cell death pathways contributes to determining TB outcomes (Araújo-Pereira and Andrade, 2025; Mohareer et al., 2018). Cytokines further shape these processes, orchestrating both innate and adaptive responses. Th1 profile cytokines (IL-2, IL-6, IL-12, IFN-γ, TNF-α) supports bacterial clearance, whereas anti-inflammatory cytokines such as IL-10 and TGF-β can dampen inflammation but may favour bacterial persistence (Aiello et al., 2023). Despite the central role of these immune mediators, the unspecific effects of INH on ROS production and cytokine responses in granulocytes and monocytes remain largely unexplored, particularly when comparing healthy individuals and LTBI individuals.

The balance between protective immunity and collateral damage is a critical aspect of TB pathogenesis, where unregulated inflammation may exacerbate lung injury and contribute to disease progression (Guzmán-Beltrán et al., 2021; Kayongo et al., 2023). Given ROS duality, acting as antimicrobial effectors yet also fuelling inflammatory damage, and cytokines shaping protective versus pathological responses, understanding how INH influences these pathways is particularly relevant for LTBI treatment. We therefore investigated the impact of physiologically relevant concentrations of INH on ROS production and cytokine secretion in circulating neutrophils, eosinophils, and monocytes. To assess ROS generation *ex vivo*, we employed an established whole-blood assay (Pioch and Blomgran, 2022) using heparinized blood, which combines the ROS-sensitive probe dihydrorhodamine 123 with cell surface antibody staining to quantify ROS in specific immune cell subsets by flow cytometry. This approach is straightforward, reproducible, and minimally disruptive, enabling the evaluation of innate immune cell function in a physiologically relevant context. By comparing healthy individuals and individuals with LTBI, this study aimed to uncover potential immunomodulatory effects of INH on innate immune cells that may influence TB pathogenesis and treatment outcomes.

## 2 Materials and methods

### 2.1 Ethics statement

Blood samples were obtained from heparinized peripheral human blood from either healthy donors or latent tuberculosis infected (LTBI) individuals from the Linköping University Hospital. Healthy donors (Linköping University Hospital Blood Bank) had given written consent for research use of the donated blood. Blood donation is classified as negligible risk to the donors and only anonymized samples were delivered to the researchers in accordance with the Declaration of Helsinki, not requiring a specific ethical approval according to paragraph 4 of Swedish law (2003:460) on Ethical Conduct in Human Research. For LTBI individuals, written informed consent was obtained, and ethical approval was obtained from the ethical committee (EPN 2021-06520-02). LTBI individuals were recruited at the Department of Infectious Disease, Linköping University Hospital, between August 2024 and May 2025 and were between 21 and 64 years old. They were tested using the QuantiFERON®-TB Gold Plus (QFT-Plus) kit (Qiagen, reference 622130). QFT-Plus has two distinct TB antigen tubes: TB Antigen Tube 1 (TB1) and TB Antigen Tube 2 (TB2). Both tubes contain peptide antigens from the MTB-complex-associated antigens, ESAT-6 and CFP-10, designed to elicit CMI responses from CD4^+^ T-helper lymphocytes. All individuals were recruited based on a positive QFT-Plus test, i.e., at least one of the QFT level ≥0.35 IU/mL.

### 2.2 Isoniazid preparation

Isoniazid is rapidly absorbed after oral administration, with peak plasma concentrations occurring within 1–2 h in fasting individuals. Reported plasma levels typically range from 3 to 6 μg/mL after a standard 300 mg daily dose, and from 9 to 15 μg/mL after a 900 mg biweekly dose (Egelund et al., 2015). To reflect clinically relevant exposure, three concentrations of INH were selected: 2 μg/mL (sub-therapeutic), 4.5 μg/mL (within therapeutic range), and 10.5 μg/mL (upper therapeutic range). The isoniazid stock solution was prepared by dissolving isoniazid powder (reference I3377-5G, Sigma-Aldrich, lot #MKCL5688) in NaCl at a concentration of 2 mg/mL. Three dilutions were then prepared in NaCl at concentrations of 200 μg/mL, 450 μg/mL, and 1,050 μg/mL. Equal volumes of each dilution were added to blood at a 1:100 ratio to achieve final concentrations of 2 μg/mL, 4.5 μg/mL and 10.5 μg/mL respectively.

### 2.3 Intracellular ROS production assay

ROS production was assessed as previously described (Pioch and Blomgran, 2022). Briefly, sodium heparin (Na-heparin) whole blood was pre-incubated with different concentrations of INH for 10 min at room temperature. 100 μL of blood was then transferred to a FACS tube on ice for 10 min and stimulated with 20 µL of either NaCl, fMLP, *E. coli*, or PMA. fMLP is a bacterial chemotactic peptide considered a relatively weak inducer of ROS, activating neutrophils through receptor-mediated signalling. *E. coli* stimulates ROS production mainly through phagocytosis, while PMA acts as a non-physiological, direct activator of NADPH oxidases and is widely used to elicit the full ROS-producing capacity of neutrophils. Dihydrorhodamine 123 (DHR-123), a membrane-permeable dye oxidized by ROS, was then added to enable the detection of intracellular ROS. Samples were incubated for 20 min in a water bath at 37 °C. To stop the reaction, tubes were placed on ice for 5 min. The antibody mix (CD125-PE from BD Biosciences; CD193-PerCP/Cyanine5.5, HLA-DR-APC, CD14-BrilliantViolet 421, and CD16-BrilliantViolet 510 from BioLegend; Fixable Viability Dye eFluor780 from ThermoFisher Scientific; and Brilliant Stain Buffer from BD Biosciences) was then added, followed by incubation for 10 min on ice and an additional 20 min at room temperature. Red blood cells were lysed, and after two washes, cells were resuspended in 200 µL of FACS buffer and acquired by flow cytometry. The percentage of DHR-123^+^ cells and the mean fluorescence intensity (MFI) of DHR-123 were determined for each cell population (see gating strategy in Supplementary Figure S1). Neutrophils were defined as SSC^high^/CD16^bright^/HLA-DR^-/low^, and eosinophils as SSC^high^/CD16^low^/HLA-DR^low/-^, CD193^+^, CD125^+^. Data from eosinophils stimulated with PMA were excluded from analysis due to marked phenotypic alterations. Monocyte subsets were identified as follows: classical monocytes (SSC^intermediate^/HLA-DR^+^/CD14^+^/CD16^-^), intermediate monocytes (SSC^intermediate^/HLA-DR^+^/CD14^+^/CD16^+^), and non-classical monocytes (SSC^intermediate^/HLA-DR^+^/CD14^low^/CD16^+^). We herein focused our analysis on classical monocytes as they are present in reliable numbers for proper analysis of intracellular ROS production. Moreover, there were no significant differences in monocyte subset distribution between healthy donors and LTBI patients (Supplementary Figure S2).

### 2.4 Phagocytosis assay

Phagocytic activity was assessed using the same protocol as for the ROS production assay, as the DHR-123 probe can be substituted with a fluorescent prey to analyse phagocytosis in the same cell subsets (Pioch and Blomgran, 2022). Briefly, sodium-heparinized whole blood was pre-incubated with different concentrations of INH for 10 min at room temperature. 100 μL of blood was then transferred to FACS tubes and placed on ice for 10 min. *Escherichia coli* (K-12 strain) BioParticles, Alexa Fluor 488 conjugate (Invitrogen, ref. E13231), were then added at a final concentration of 0.02–0.04 particles/mL, and samples were incubated for 20 min in a 37 °C water bath. The reaction was stopped by placing the tubes on ice for 5 min. Antibody staining was then performed by adding the same antibody mix as for the DHR-123 assay, followed by incubation for 10 min on ice and 20 min at room temperature. Red blood cells were lysed, and samples were washed twice before resuspension in 200 µL of FACS buffer. Data were acquired on a flow cytometer, and for each cell population, the percentage of Alexa Fluor 488^+^ cells and the mean fluorescence intensity (MFI) were determined using the same gating strategy as for the ROS assay.

### 2.5 Plasma isolation

To retrieve plasma, 400 µL of whole blood was centrifuged at 2,000 × g for 15 min at 4 °C. Plasma was isolated at 0 h and after 24 h incubation of unstimulated blood, or with isoniazid at final concentrations of 2 μg/mL, 4.5 μg/mL and 10.5 μg/mL. The supernatant was then collected and stored at −80 °C until further processing.

### 2.6 Cytokines analysis

Cytokine levels were measured in plasma samples from 7 healthy controls and 6 LTBI individuals. Concentrations of TNF, IL-8, IL-6, IL-1β, IFN-γ, TGF-β, and IL-10 were determined using a cytometric bead array (CBA), according to the manufacturer’s instructions (BD Biosciences).

### 2.7 Flow cytometry

All work was performed on a Gallios flow cytometer (Beckman Coulter, Brea, CA) equipped with a 405 nm, 488 nm, and a 638 nm laser allowing detection of 10 colours. FlowJo v10 was used for data analysis.

### 2.8 Statistics

Statistical analyses were performed using GraphPad Prism version 10.5.0. Normality was assessed using the Shapiro-Wilk test. Depending on the distribution, significance testing was carried out using either a parametric test (one-way ANOVA) or a non-parametric test (Friedman test followed by Dunn’s *post hoc* test). For ROS production, comparisons were made for each stimulation condition separately, testing each INH concentration against the vehicle control (NaCl). Multiple statistical analyses were structured around predefined biological comparisons. Indeed, ROS production under specific stimulation conditions and INH concentrations was assessed within each cell type using paired analyses within individuals. For cytokine levels, paired analyses were performed comparing the vehicle control after 24 h with all other plasma conditions, separately for healthy and LTBI subjects. In addition, healthy controls and LTBI individuals were compared for each plasma condition. A p-value <0.05 was considered statistically significant.

## 3 Results

### 3.1 Isoniazid differentially affects ROS production in circulating neutrophils, eosinophils, and monocytes in healthy subjects

**FIGURE 1:**
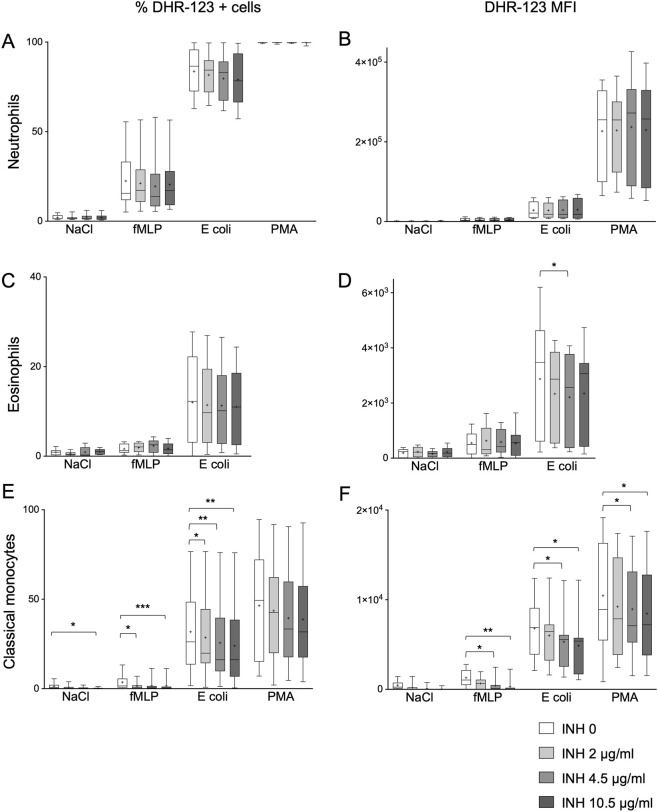
Percentage of DHR-123+ cells (left column) and DHR-123 MFI (right column) for healthy subjects (n = 9). Box shows 25–75 percentiles; whiskers show 10–90 percentiles, for neutrophils **(A,B)**, eosinophils **(C,D)**, and classical monocytes **(E,F)**. The line in the middle of the box is plotted at the median, “+” represent the mean. Paired measures one-way ANOVA between INH 0 and every other antibiotic concentration for each stimulation separately, Tukey’s post-test. *p < 0.05, **p < 0.01 and ***p < 0.001.

Intracellular ROS production was evaluated in neutrophils, eosinophils, and classical monocytes following stimulation with N-formylmethionyl-leucyl-phenylalanine (fMLP), *E. coli* (*E. coli*), or phorbol myristate acetate (PMA), in the presence of increasing concentrations of isoniazid. In neutrophils, fMLP induced a modest response, *E. coli* elicited a stronger response, and PMA triggered the maximal ROS production in all neutrophils (Figure 1A), with a markedly increased DHR-123 MFI (Figure 1B). ROS production was unaffected by isoniazid at any of the concentrations tested.

Following *E. coli* stimulation, INH did not induce a change in the frequency of ROS-producing eosinophils across the tested concentrations (Figure 1C). However, a significant reduction in DHR-123 MFI was observed at 4.5 μg/mL compared with untreated controls (p = 0.041), with no additional significant differences at higher concentrations (Figure 1D). In classical monocytes, isoniazid induced a more pronounced inhibitory effect. The percentage of ROS-producing cells decreased with increasing concentrations of isoniazid across all stimulation conditions (Figure 1E), accompanied by a significant reduction in DHR-123 MFI values (Figure 1F). To determine whether the decrease in ROS production when stimulated with *E. coli* was due to altered bacterial uptake rather than a direct effect on oxidative activity, a phagocytosis assay using fluorescently labelled *E. coli* was performed. The percentage of phagocytic cells and the mean fluorescence intensity of internalized bacteria remained unchanged across all INH concentrations, indicating that the reduced ROS signal was not a consequence of impaired phagocytosis (Supplementary Figure S3).

### 3.2 Socio-demographic and clinical characteristics of LTBI individuals

LTBI individuals were recruited between August 2024 and May 2025 (Table 1). The participants ranged from 21 to 64 years old and were born in various countries, including Somalia (n = 2), Uganda (n = 2), Sweden, the Philippines, Afghanistan, Iraq, and Romania. Two individuals were BCG vaccinated, three were not, and four had unclear vaccination histories. Indications for testing included pregnancy screening (n = 3), planned immunosuppression (n = 3), high-endemic country origin, investigation for gastrointestinal bleeding, and suspected active TB due to erythema nodosum. A total of 9 individuals were included as LTBI, based on the QuantiFERON-TB assay, including 6 females, 2 males, and 1 individual born with both sexes. All individuals had a positive QFT test and QFT TB1 levels ranged from 0.5 IU/mL to 8.3 IU/mL. QFT TB2 levels ranged from 0.16 IU/mL to 7.87 IU/mL.

**TABLE 1:** Socio-demographic and clinical characteristics of study participants.

Subject	Sex	Age	Country of birth	BCG vaccinated	Indication for testing	QFT TB1 level	QFT TB2 level
LTBI 1	M	62	Somalia	Yes	Gastrointestinal bleeding	1,4	2,09
LTBI 2	F	51	Sweden	Yes	Planned immunosuppression	1,6	1,53
LTBI 3	F	35	The Philippines	Unclear	Pregnancy screening	0,5	0,16
LTBI 4	F	28	Somalia	Unclear	Pregnancy screening	2,6	2,67
LTBI 5	F	60	Afghanistan	No	Erythema nodosum	1,7	1,78
LTBI 6	M	57	Iraq	No	Planned immunosuppression	3,9	5,68
LTBI 7	F	64	Romania	No	Planned immunosuppression	8,3	7,64
LTBI 8	F	31	Uganda	Unclear	Pregnancy screening	1,7	1,23
LTBI 9	I	21	Uganda	Unclear	High-endemic country	5,8	7,87

QFT levels are expressed in IU/mL. M: male, F: female, I: intersex.

### 3.3 Effect of INH on ROS production in LTBI individuals

**FIGURE 2:**
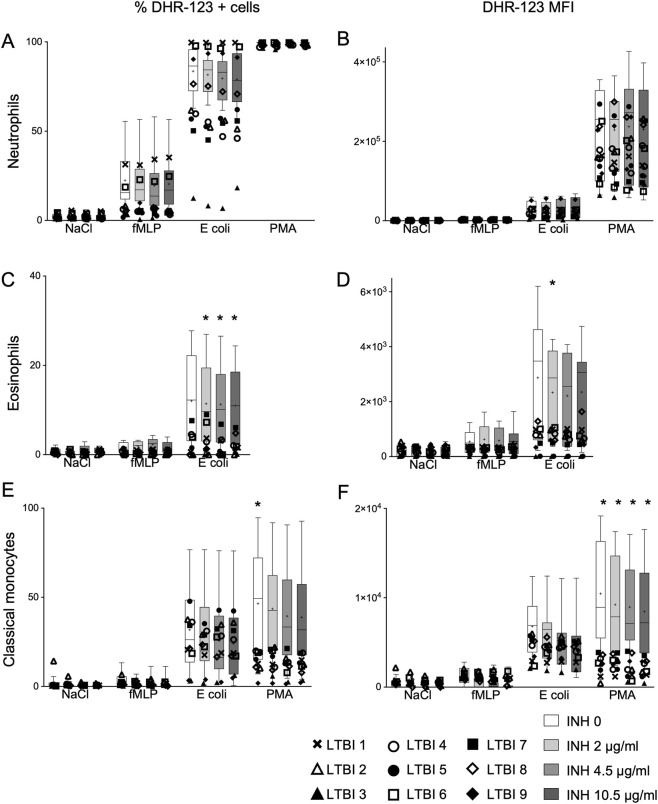
Percentage of DHR-123+ cells (left column) and DHR-123 MFI (right column) for healthy subjects (bars in grey, n = 9) and LTB patients (symbols, n = 9). Box shows 25-75 percentiles; whiskers show 10-90 percentiles, for neutrophils **(A,B)**, eosinophils **(C,D)**, and classical monocytes **(E,F)**. The line in the middle of the box is plotted at the median, “+” represent the mean. Unpaired measures one-way ANOVA between healthy and LTBI subjects for each antibiotic concentration and each stimulation separately, Tukey´s post-test. *p < 0.05.

In LTBI individuals, ROS production was assessed using the same assay as in healthy individuals. In neutrophils, the percentage of DHR-123^+^ cells was comparable between LTBI individuals and healthy controls under unstimulated conditions or after PMA stimulation. In contrast, following fMLP or *E. coli* stimulation, ROS responses in LTBI individuals displayed greater inter-individual variability and tended to be lower overall compared with healthy controls. Median ROS levels across all INH concentrations were consistently below the 25th percentile of the healthy cohort (for fMLP, median was 5.7, 5.5, 6.8 and 5.4 vs. 18.0, 16.6, 16.9 and 18.5 in controls; for *E. coli*, median was 61.7, 55.2, 57.2 and 62.1 vs. 87.8, 87.5, 84.0 and 80.4 in controls; for increasing INH concentrations respectively) (Figure 2A). Interestingly, the two male subjects of the cohort showed responses within the control range (LTBI 1 and LTBI 6) and as an exploratory analysis excluding these subjects, the remaining LTBI individuals exhibited a diminished proportion of ROS-producing neutrophils compared to healthy controls upon stimulation with fMLP, with a significant decrease for cells without INH and with 2 μg/mL of INH (p = 0.029 and p = 0.049 respectively).

When stimulated with *E. coli*, one additional subject (LTBI 9, intersex and second highest TB1 and TB2 levels) also showed a higher response, while one patient (LTBI 3, with negative/low QFT values) displayed a particularly low response (Figure 2A). The remaining LTBI individuals exhibited a reduction in the proportion of ROS-producing neutrophils upon stimulation with *E. coli*. The DHR-123 MFI in neutrophils from LTBI individuals remained within the range observed in healthy controls across all INH concentrations and stimuli (Figure 2B). Interestingly, the individual with negative/low QFT values (LTBI 3) showed the lowest response when stimulated with *E. coli* or PMA.

In eosinophils, both the proportion of DHR-123^+^ cells and the corresponding MFI were comparable between LTBI individuals and healthy controls under unstimulated or fMLP-stimulated conditions. However, following *E. coli* stimulation, LTBI individuals exhibited lower oxidative responses. In LTBI individuals, the percentage of DHR-123^+^ eosinophils significantly decreased compared to healthy controls at each corresponding isoniazid concentration including the no-drug condition, while the MFI was only significantly reduced at the lowest INH concentration (Figures 2C,D). For appreciating the relative difference between the LTBI individuals and the healthy controls in terms of *E. coli*-stimulated ROS-induction in eosinophils, median values from all doses ±INH were aggregated, showing 11.3% vs. 2.0% DHR+, for controls vs. LTBI, with p < 0.0001).

In classical monocytes, PMA stimulation elicited a reduced oxidative response in LTBI individuals compared with healthy controls, whereas responses under unstimulated, fMLP-, or *E. coli*-stimulated conditions remained within the control range (Figures 2E,F). The proportion of DHR-123^+^ monocytes with PMA stimulation was significantly lower in LTBI individuals compared to healthy controls in the absence of isoniazid, while the MFI was significantly reduced at each corresponding INH concentrations in LTBI individuals compared to healthy controls. Similarly, aggregated median values from all doses ±INH for PMA-stimulated classical monocytes the difference in DHR+ cells were 41.5% vs. 12.2% DHR+, for controls vs. LTBI, and had a p-value <0.0001.

Across all cell types and stimulation conditions, no consistent effect of INH concentrations was observed in LTBI individuals, except for a decrease in DHR-123 MFI in eosinophils between 0 and 4.5 μg/mL of INH under fMLP stimulation (p = 0.0405).

### 3.4 Effect of INH on plasma cytokine levels in LTBI individuals and healthy controls

**FIGURE 3:**
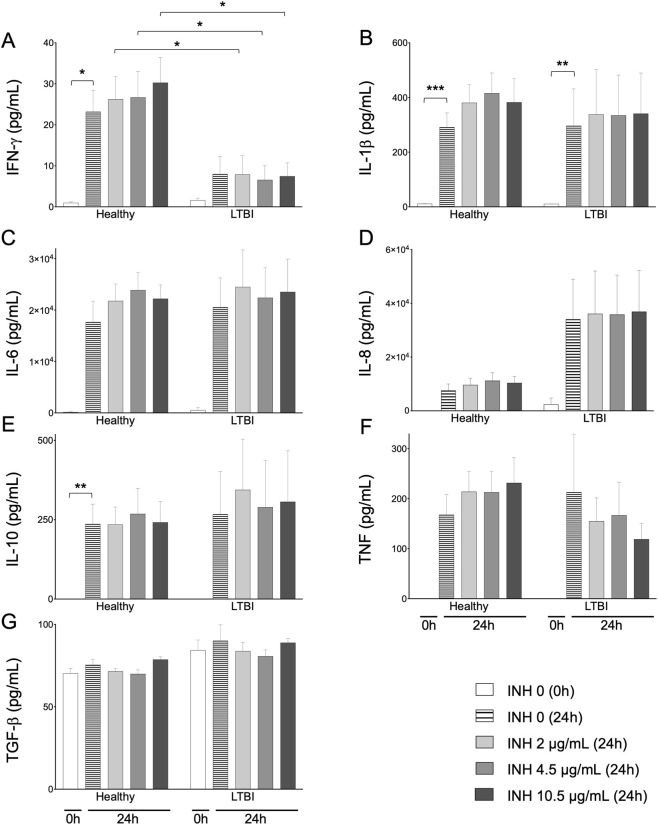
Concentration (in pg/mL) of pro- or anti-inflammatory cytokines in healthy subjects (n = 7) and LTBI patients (n = 6). The level of IFN-γ **(A)**, IL-6 **(B)**, IL-8 **(C)**, IL-10 **(D)**, IL-1β **(E)**, TNF **(F)** and TGF-β **(G)** were determined in plasma at t = 0 and t = 24h, without stimulation or with 2 μg/mL, 4.5 μg/mL or 10.5 μg/mL of isoniazid in blood. Data presented as mean ± SD. Statistical comparisons were made using a paired ANOVA test comparing every condition to the 24h incubation without isoniazid. *p < 0.05, **p < 0.01 and ***p < 0.001.

In healthy donors, IFN-γ levels increased significantly after 24 h compared to baseline, but exposure to INH did not alter IFN-γ secretion. In contrast, plasma IFN-γ levels in the LTBI group were significantly lower than that in healthy controls and remained low and unchanged across all INH concentrations (Figure 3A). For IL-1β, spontaneous cytokine production between 0 and 24 h in the absence of stimulation was significant in both groups, with no differences between healthy and LTBI subjects (Figure 3B). Similar trends were observed for IL-6, IL-8, IL-10, and TNF, although not always statistically significant (Figures 3C–F). TGF-β concentrations remained stable in both groups at both time points (Figure 3G). Notably, for all cytokines except IL-8 and TGF-β, one LTBI donor (LTBI 9), who exhibited the second highest QFT-TB1 and TB2 values, also showed the highest cytokine levels. Collectively, these findings indicate that INH did not significantly influence cytokine concentrations under the tested conditions.

## 4 Discussion

This study primarily aimed to determine whether INH has an unspecific effect on ROS production in innate immune cells and whether this effect differs between healthy and LTBI individuals. The findings reveal that INH dose-dependently dampens ROS production in classical monocytes from healthy donors, while leaving neutrophil responses unaffected. Intriguingly, this modulatory effect by INH was absent in LTBI individuals, showing significantly and overall markedly reduced ROS responses compared to healthy controls, suggesting an altered redox steady state associated with latent infection. Together, these results provide new insight into the unspecific properties of INH and help clarifying its immunomodulatory capacity beyond its direct antimycobacterial activity.

ROS are central effectors in innate immunity. They contribute to bacterial killing, yet excessive ROS can damage host tissues and trigger necrotic cell death of phagocytes, which both may favour Mtb dissemination (Ehrt and Schnappinger, 2009; Dallenga et al., 2017). Mtb has evolved defence mechanisms to counteract oxidative stress (Voskuil et al., 2011; Trivedi et al., 2012), which complicates the balance between microbial control and host damage. INH has been reported to exert cytoprotective effects against ROS-induced necrosis, possibly by enhancing ATP biogenesis and supporting mitochondrial homeostasis (Khan et al., 2016a; Khan et al., 2016b). However, the present study did not explore any biological mechanism and our data are therefore limited to demonstrating that INH reduces ROS generation in circulating monocytes from healthy donors. While this finding indicates that INH can modulate oxidative responses in innate immune cells, the underlying mechanisms remain undefined.

INH selectively affecting monocytes but not neutrophils is intriguing, since both cell types rely on ROS as part of their antimicrobial arsenal. Monocytes and neutrophils rely on distinct pathways for ROS generation, with monocytes producing lower, sustained levels through NADPH oxidase and mitochondria, while neutrophils can also generate rapid bursts that involve myeloperoxidase to enhance the microbicidal effect of ROS. This could explain why monocytes are more susceptible to pharmacological interference by INH. The altered baseline in LTBI individuals could mask pharmacological modulation and suggests that INH’s immune-metabolic effects is less pronounced in the very population that receives therapy.

Several studies have demonstrated that cytokines such as IFN-γ, TNF, and IL-1β contribute to immune control in LTBI (Kudryavtsev et al., 2024). It is therefore likely that pre-existing cytokine activity in LTBI individuals influences how circulating innate immune cells respond to INH. In our cohort, IFN-γ levels in plasma were lower in LTBI subjects compared to healthy donors for the three doses of INH tested. Although the mechanisms underlying this difference remain unclear, reduced baseline IFN-γ signalling could reflect lower cell priming, which may in turn affect oxidative responses. Our findings extend previous work done by Kielland et al., who used isolated human polymorphonuclear neutrophils from healthy donors incubated with subtherapeutic, therapeutic, and supratherapeutic concentrations of INH (Kielland et al., 2011). In that *ex vivo* PMN model, oxidative burst was attenuated only at the supratherapeutic concentration, whereas our results show measurable inhibition at clinically relevant doses in circulating human innate immune cells. The absence of INH-dependent cytokine modulation observed under the tested conditions, namely, incubation of unstimulated whole blood for 24 h with INH at 2, 4.5, or 10.5 μg/mL and analysis of cytokines in the resulting plasma, is consistent with previous reports describing limited proinflammatory effects of INH. In Mtb-infected murine bone marrow–derived macrophages, INH treatment reduced the secretion of TNF, IL-6, and IL-1β (Kim et al., 2012), while in a human *in vitro* granuloma model reflecting active TB, INH did not alter production of IL-1β or TNF (Yamashiro et al., 2016). Reports describing reduced immune cell functionality in other contexts (Stassar et al., 1994; De Boer et al., 1992) also fit our observation of reduced oxidative activity, though our data suggest this suppression is selective for monocytes in healthy subjects rather than global. Taken together, these findings strengthen the view that INH acts on redox modulation rather than broad immune inhibition.

From a therapeutic perspective, lowering excessive ROS during TB infection may protect host tissues and limit necrosis-driven bacterial spread, particularly in early infection or inflammatory environments. However, attenuating ROS too much could theoretically compromise bactericidal functions in individuals with otherwise intact redox responses. Our results in eosinophils and classical monocytes from LTBI individuals suggest that ROS production is lower than in healthy controls, and INH does not cause further suppression. While this could be advantageous in avoiding excessive immune dampening, we cannot draw conclusions about ROS activity at the infection site.

Regarding high-dose INH, it appears to retain activity in drug-resistant tuberculosis, although the mechanisms remain incompletely understood. Elevated INH concentrations can partially overcome resistance by maintaining inhibition of the type II fatty acid synthase (FAS-II) pathway, as shown in a randomized trial where doses of 10–15 mg/kg achieved bacterial killing in inhA-mutated isolates, particularly among slow and intermediate N-acetyltransferase 2 acetylators (Gausi et al., 2021). Experimental data similarly indicate residual FAS-II inhibition even in highly resistant strains (Fu and Shinnick, 2007), though responses are attenuated. It may also be due to reduced immunopathology, as high-dose INH can induce apoptosis in activated CD4^+^ T cells and dampen excessive antigen-specific responses (Tousif et al., 2014), with complementary studies suggesting improved lung outcomes when paired with immunomodulatory strategies (Koo et al., 2011). A systematic review of 5,103 subjects with multi-drug resistant TB (MDR-TB) reported a 76.5% treatment success rate with high-dose INH (>300 mg/day or >5 mg/kg/day) (Zhou et al., 2024). Overall, while evidence supports dose-dependent efficacy even in case of resistance, the contribution of immunomodulatory effects remains to be fully elucidated.

A notable strength of this study is the use of a straightforward, minimally invasive, whole-blood assay, which preserves physiological interactions between immune cells and plasma components. The testing of clinically relevant INH concentrations further enhances translational value. However, only short-term exposures were examined, and chronic exposure could yield distinct effects on cell activation. The *in vitro* design also does not account for pharmacokinetic processes, hepatic metabolism, or long-term immune adaptation. In addition, experiments were performed on circulating granulocytes and monocytes, which do not fully reflect immune responses at the site of infection, such as in pulmonary tissue or granulomas. While circulating neutrophils and monocytes represent the pool of cells recruited to infected tissues and systemic immune status can influence local responses, these results are not indicators of *in situ* tuberculosis immunity.

While reduced ROS responses were observed in cells from LTBI individuals compared to healthy controls, this study is descriptive and does not establish a mechanistic basis for these differences. Several explanations may underlie the observed phenotype, including exhaustion, systemic inflammatory status, nutritional factors, or prior subclinical exposure to *M. tuberculosis*. These parameters were not assessed here and may independently shape oxidative responses. The LTBI cohort was heterogeneous regarding age, geographic origin, BCG vaccination status, and magnitude of QFT responses. Larger studies with systematic control for demographic and immunological variables will be required to determine the contribution of these factors. Moreover, sex differences in tuberculosis epidemiology and immune responses are well established, with males exhibiting higher TB incidence and mortality globally (Dabitao et al., 2023). Although our cohort was not powered to evaluate sex as a biological variable, the observation that male participants exhibited higher ROS responses for neutrophils under certain stimulations warrant future studies designed to address sex-dependent modulation of oxidative responses during TB treatment.

Overall, our data indicate that INH can modulate monocyte ROS production independently of its antimicrobial activity, even within therapeutic concentration ranges. While this effect appears muted in LTBI individuals, its selective and dose-dependent nature suggests that INH may subtly modulate redox signalling without affecting overall immune competence. Understanding such host-directed aspects of TB pharmacotherapy could inform strategies to balance antimicrobial efficacy with the preservation of host immune resilience.

## Data Availability

The original contributions presented in the study are included in the article/Supplementary Material, further inquiries can be directed to the corresponding author.
