# Supplementary material for: Differential regulation of monocyte oxidative burst by isoniazid in healthy and latent tuberculosis-infected subjects
Source: Front Pharmacol. 2026 Mar 31;17:1763287. doi: 10.3389/fphar.2026.1763287 (PMC13076521; doi:10.3389/fphar.2026.1763287)
Supplement: Supplementary file 1 [file Supplementaryfile1.docx]

Supplementary Material


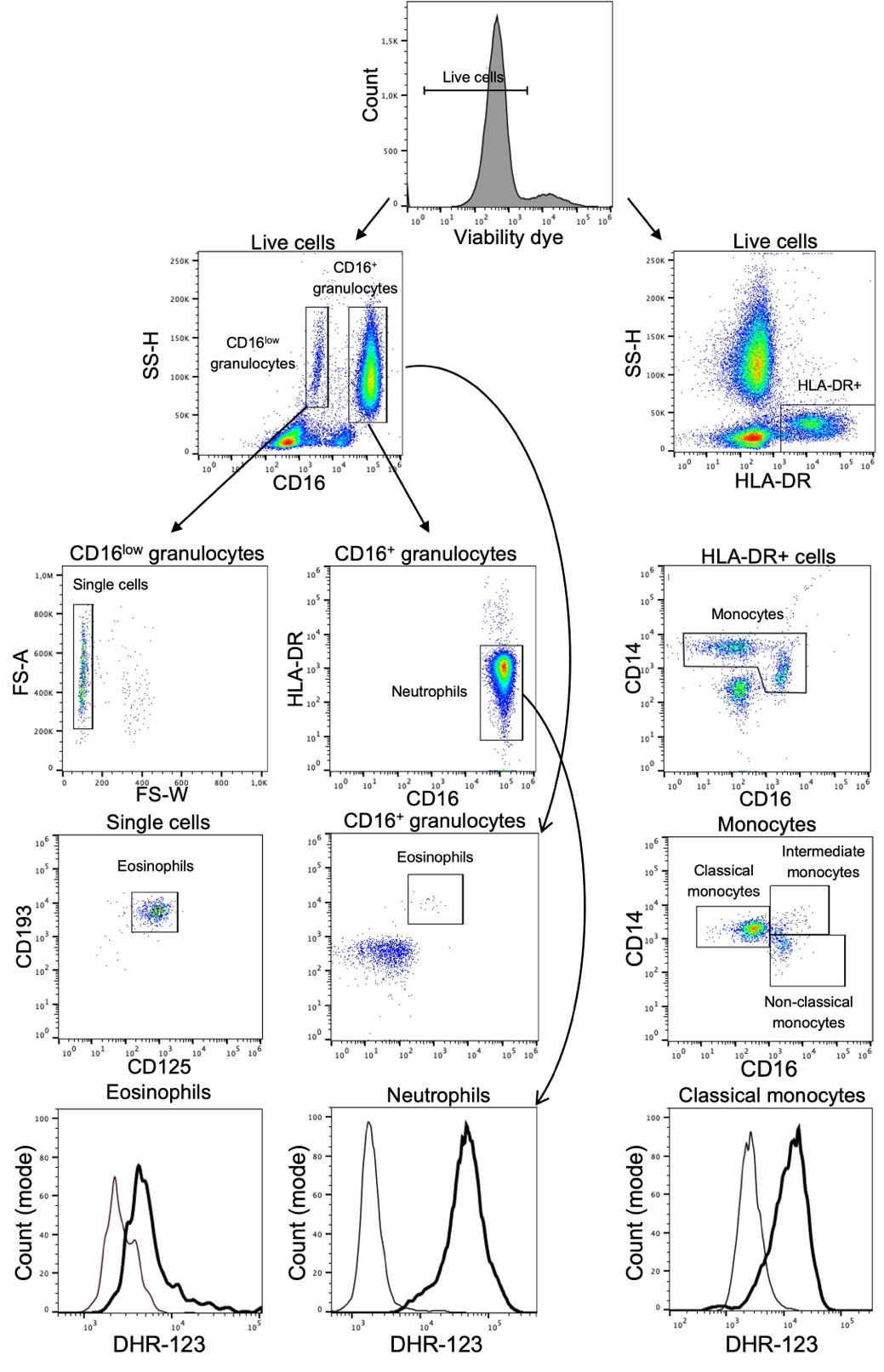


**Supplementary Figure 1.** **Gating strategy for neutrophils, eosinophils and classical monocytes.** On histograms, the thin line represents the ice control, and the bold line is from blood stimulated with *E. coli*, without INH.


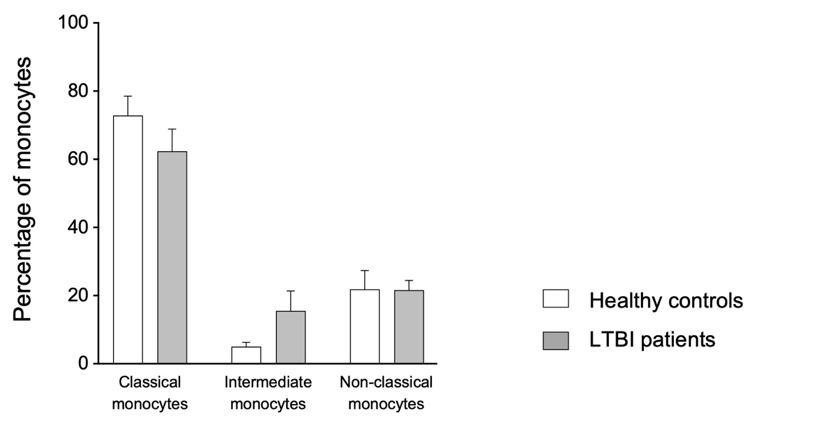


**Supplementary Figure 2. Monocytes subsets distribution in healthy donors and LTBI patients.** Data are presented as mean ± SEM for cells incubated without INH in whole blood from n=9 independent donors for both groups.


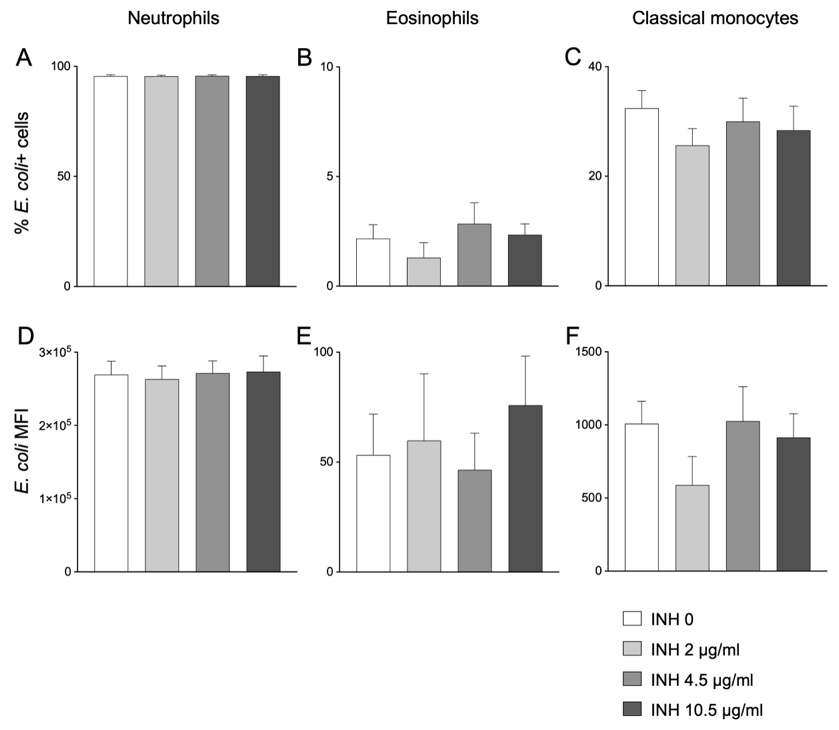


**Supplementary figure 3. Phagocytosis of *E. coli* by circulating immune cell subsets in healthy donors in the presence of isoniazid.** Neutrophils (A and D), eosinophils (B and E), and monocytes (C and F) from whole blood were incubated with AF488-labeled E. coli, and phagocytosis was assessed by flow cytometry. The percentage of AF488-positive cells (A, B and C) and the mean fluorescence intensity (MFI) (D, E and F) were quantified for each cell type. Data are presented as mean ± SEM for cells incubated without INH (0 µg/mL) or with increasing concentrations of INH (2, 4.5, and 10.5 µg/mL) in whole blood from n=9 independent donors. A fluorescence threshold was defined using an ice control (cells incubated with AF488-labeled E. coli on ice for 20 min), in which ≤0.5% of cells were positive; the MFI of this sample was used as background and subtracted from the values shown.
